# Myogenic Fusogen-Engineered Lipid Nanoparticles Enhance mRNA Delivery in Skeletal Muscle

**DOI:** 10.1002/adfm.78165

**Published:** 2026-09-12

**Authors:** Fangyu Zhang, Zhisheng Xu, Yaou Duan, Yehui Sun, Shiying Wu, Tao Long, Mateusz Durbacz, Li Zhang, Yufen Xiao, Yu-Chung Pien, Erick D. Guerrero, Zeru Tian, Xu Wang, Pratima Basak, Sumanta Chatterjee, Ning Liu, Xiaochun Li, Eric N. Olson, Daniel J. Siegwart

**Affiliations:** 1 Department of Biomedical Engineering, Department of Biochemistry, Simmons Comprehensive Cancer Center, Program in Genetic Drug Engineering, University of Texas Southwestern Medical Center, Dallas, Texas, USA; 2 Department of Molecular Biology, University of Texas Southwestern Medical Center, Dallas, Texas, USA; 3 Department of Molecular Genetics, University of Texas Southwestern Medical Center, Dallas, Texas, USA; 4 Department of Physiology, University of Texas Southwestern Medical Center, Dallas, Texas, USA; 5 Department of Biophysics, University of Texas Southwestern Medical Center, Dallas, Texas, USA; 6 Hamon Center for Regenerative Science and Medicine, University of Texas Southwestern Medical Center, Dallas, Texas, USA

**Keywords:** cell biology, gene delivery, lipid based nanoparticles, messenger rna, skeletal muscle, transfection

## Abstract

Lipid nanoparticles (LNPs) have emerged as powerful vehicles for mRNA therapeutics, yet efficient delivery to skeletal muscle remains challenging. Here, we engineer LNPs by integrating Myomaker, a muscle-specific fusogen, onto their surface to enhance skeletal muscle delivery. Optimized Myomaker-LNP (Mymk-LNP) formulations demonstrated high fusogen integration, uniform size, and efficient mRNA encapsulation. Mymk-LNPs exhibited selective in vitro transfection in differentiating myocytes while minimizing uptake in myoblasts and fibroblasts. Systemic and local administration of Mymk-LNPs in vivo led to significantly increased mRNA delivery to skeletal muscle, particularly in models with muscles undergoing fusion, such as developing muscles in neonatal mice and chemically injured muscles in adult mice. Furthermore, Mymk-LNPs mediated efficient Cre mRNA-mediated DNA recombination in injured skeletal muscle as quantified by activation of a Cre-dependent tdTomato reporter. These findings highlight the potential of fusogen-engineered LNPs as a targeted and effective platform for delivery of mRNA therapeutics to muscle.

## 1 ∣ Introduction

Following the success of mRNA-based COVID-19 vaccines, therapeutic messenger RNA (mRNA) approaches hold great promise to enable precise and transient protein expression for the treatment of genetic disorders, metabolic diseases, and cancer [1–10]. Lipid nanoparticles (LNPs) have emerged as the most effective non-viral mRNA delivery platform, providing high cellular uptake, efficient endosomal escape, and robust transfection efficiency [11–13]. However, following systemic administration, conventional LNPs predominantly accumulate in the liver due to interactions with serum proteins such as apolipoprotein E (ApoE), thereby limiting their application for extrahepatic targets such as skeletal muscle [14, 15]. Skeletal muscle represents a critical therapeutic target for treating genetic disorders, metabolic syndromes, and tissue regeneration [16–20]. While viral vectors, particularly adeno-associated viruses (AAVs), have been widely used for muscle gene therapy, their limited transgene capacity, high immunogenicity, and long-term genome integration pose significant risks and challenges to clinical translation [21–25].

Recent LNP engineering strategies, including modification of lipid compositions for endogenous targeting and conjugation of targeting ligands such as antibodies or peptides for active targeting [12], have demonstrated enhanced nanoparticle biodistribution toward extrahepatic tissues such as lungs, spleen, bone marrow, and brain [26–30]. However, achieving selective and efficient mRNA delivery to skeletal muscle remains a major challenge [31]. Therefore, developing next-generation LNP formulations that enhance muscle-specific transfection while minimizing off-target effects is crucial for advancing mRNA-based muscle therapies.

Skeletal muscle possesses a unique regenerative capacity, relying on fusion of satellite cells for growth, repair, and homeostasis [32–34]. Myomaker, a transmembrane protein expressed exclusively in myogenic cells, plays a fundamental role in this process by mediating cell-cell fusion [35, 36]. Typically, Myomaker expression is tightly restricted to a defined temporal window during muscle development and regeneration [35, 37]. As a result, Myomaker on both opposing membranes mediates efficient membrane fusion [36, 38]. This essential function in membrane remodeling during muscle development and regeneration makes Myomaker a promising candidate for enhancing skeletal muscle-selective mRNA delivery as recently shown for lentiviruses and virus-like particles bearing Myomaker on the surface [39, 40]. Inspired by these biomimetic design approaches, we hypothesized that functionalizing LNPs with full-length Myomaker could facilitate targeted fusion with myofibers by mimicking the natural myoblast fusion process, enhancing intracellular mRNA uptake and translation. This strategy aligns with recent advancements in proteolipid vehicles, where surface-modified fusion proteins on liposomes or LNPs leverage mechanisms of fusogenic viruses to improve DNA or RNA delivery and cellular uptake in multiple organs [41, 42]. Compared to recent advancements in muscle-targeting LNPs conjugated with fusogen-derived peptides, we speculated that incorporating LNPs with full-length Myomaker might not only overcome limitations associated with passive LNP accumulation, but also provide an innovative fusion-driven strategy for delivering therapeutic mRNA to regenerating skeletal muscle while reducing unintended uptake by non-muscle tissues [43].

In this study, we developed Myomaker-functionalized LNPs (Mymk-LNPs) as a platform for muscle-enriched mRNA delivery and Cre-mediated DNA recombination. By optimizing Myomaker conjugation onto LNP surfaces, we achieved efficient protein integration while maintaining nanoparticle stability and high mRNA encapsulation efficiency. The optimized Mymk-LNP formulation demonstrated enhanced tdTomato mRNA transfection in myotubes. More importantly, Mymk-LNP showed selective preferential uptake in differentiating myotubes expressing Myomaker compared to myoblasts and fibroblasts with little or no Myomaker expression in vitro, supporting Myomaker-mediated fusion mechanism. To assess in vivo muscle-targeting capability, we investigated Mymk-LNP-mediated mRNA delivery in neonatal mice and adult mice following muscle injury, where systemic and local administration enhanced transfection efficiency in developing and regenerating skeletal muscle. Using Cre mRNA encapsulated in Mymk-LNPs, we observed robust Cre-dependent tdTomato reporter expression in regenerating myofibers of Ai14 mice. These findings highlight the potential of fusogen-engineered LNPs as a broad platform for targeted mRNA therapeutics in regenerating muscle, with potential applications for muscular dystrophies treatment, regenerative medicine, and precision genetic therapies.

## 2 ∣ Results

### 2.1 ∣ Design and Characterization of Muscle-Specific Fusogen-Engineered LNPs

To evaluate the feasibility of integrating Myomaker with LNPs, we formulated and tested lipid formulations (**LNPs 1–4**) that were previously shown to mediate hepatic and extrahepatic mRNA delivery in vivo (Figure 1a). **LNP 1** contained four primary lipid components: 2-dioleoyl-sn-glycero-3-phosphoethanolamine (DOPE), cholesterol, 1,2-dimyristoyl-sn-glycero-3-methoxypolyethylene glycol-2000 (DMG-PEG2000), and the ionizable lipid 5A2-SC8. In LNPs 2–4, additional lipids—1,2-dioleoyl-3-dimethylammonium-propane (DODAP), 1,2-dioleoyl-sn-glycero-3-phosphate (18PA), and 1,2-dioleoyl-3-trimethylammonium-propane (DOTAP)—were added in specific ratios to modulate nanoparticle properties [28]. Mouse Myomaker was expressed in Sf9 cells using the baculovirus system [36]. The proteins were then incorporated onto the surface of the LNPs using a post-incubation method [44] at a Myomaker-to-lipid weight ratio of 5%, ensuring optimal conjugation while maintaining nanoparticle stability. Protein quantification revealed that **LNP 4** achieved the highest Myomaker integration efficiency (72.0%), which was significantly higher than **LNPs 1–3** (15.3%, 15.4%, and 8.4%, respectively) (Figure 1b). Among all formulations, the DOTAP-containing **LNP 4** (denoted as “Mymk-LNP 4”) displayed the most favorable Myomaker integration efficiency and was therefore selected for the subsequent experiments. By tuning the Myomaker-to-lipid weight ratio, we found that increasing the Myomaker input in **LNP 4** resulted in a slight increase in particle size while maintaining a low polydispersity index (PDI < 0.3), indicative of uniform nanoparticles (Figure 1c). The zeta potential of each formulation was characterized to confirm changes in surface charge upon Myomaker incorporation (Figure 1d). Further analysis revealed that Myomaker protein integration efficiency was positively correlated with the input Myomaker protein-to-lipid ratio. Increasing the Myomaker-to-lipid weight ratio from 0.25% to 1% and 5% led to a modest increase in Myomaker integration efficiencies on LNPs (61.7%, 72.0%, and 79.2%, respectively) (Figure 1e), corresponding to 1543.1, 7202.7, and 39624.6 ng of Myomaker molecules per mg of nanoparticle (Figure 1f).

The presence of Myomaker on **LNP 4** was validated by western blot analysis, where Mymk-LNP 4 displayed a distinct Myomaker protein band (~21 kDa), while non-functionalized LNPs showed no detectable signal (Figure 1g). To determine whether Myomaker was integrated at the single-particle level, an immunogold labeling approach followed by transmission electron microscopy (TEM) was employed. Following anti-FLAG antibody incubation, 12 nm gold-conjugated secondary antibodies specifically labeled the N-terminal FLAG tag on Myomaker present on the surface of Mymk-LNPs. In contrast, LNPs without Myomaker exhibited no gold labeling, confirming successful and selective Myomaker incorporation (Figure 1h). Structure analysis using cryo-electron microscopy (Cryo-EM) revealed uniform spherical particles with a well-defined outer lipid bilayer (Figure 1i). Notably, this structural feature is similar to bilayer-based liposomes, which have previously been utilized for proteolipid assembly [41], potentially facilitating Myomaker incorporation and contributing to its high protein integration efficiency.

Since the native orientation of Myomaker is crucial for its function in myoblast fusion [36], we next investigated its topology on the Mymk-LNP 4 surface. Under physiological conditions, Myomaker localizes to the plasma membrane, with its N-terminal domain facing extracellularly and its C-terminal domain facing intracellularly, a configuration that it is essential for its function in myoblast fusion and muscle regeneration [36, 45]. To determine the orientation of Myomaker on the intact Mymk-LNP 4, we employed a dual-detection system using the 18G7 antibody (specific to the intracellular loop 3), that we generated previously [36] and an anti-FLAG antibody (specific to the extracellular N-terminus) (Figure S1). Fluorescence-based secondary antibody detection demonstrated strong anti-FLAG immunostaining on Mymk-LNP 4, with minimal 18G7 staining, consistent with the native orientation of Myomaker on the nanoparticle surface (Figure 1j), with the correct protein orientation facing outward from the LNP. In contrast, other LNP formulations exhibited notably fewer and weaker fluorescence signals, indicating low protein integration with random orientations (Figure S2). To ensure that incorporation of Myomaker on LNPs does not affect their colloidal stability, we incubated both LNP 4 and Mymk-LNP 4 in phosphate-buffered saline (PBS) at 4°C for a period of 7 days. Both LNP formulations showed minimal change (<7%) in their hydrodynamic sizes and maintained encapsulation efficiency above 70% over 7 days, indicating high storage stability in the buffer even after Myomaker conjugation (Figure S3). Finally, to confirm that Myomaker remains stably incorporated on **LNP 4** during delivery, we performed a serum stability study by incubating Mymk-LNP 4 in 50% mouse serum at 37°C for a period of 24 h. As shown in the Western blot in Figure S4, the amount of Myomaker associated with Mymk-LNP 4s remained comparable to that of freshly prepared Mymk-LNP 4s after serum incubation up to 24 h, supporting the stability of the Myomaker-functionalized LNP during delivery (Figure S4).

Collectively, these findings establish that Myomaker can be effectively engineered onto LNP surfaces, with Mymk-LNP 4 exhibiting the highest protein loading efficiency, stable particle size, and correct protein orientation, making it a modular platform for muscle-targeted delivery.

### 2.2 ∣ Fusogen-Engineered LNPs Improve mRNA Transfection to Myotubes

To investigate whether Myomaker functionalization enhances LNP delivery to muscle cells, Mymk-LNP 4 was labeled with the hydrophobic lipid dye, 1,1′-dioctadecyl-3,3,3′,3′ - tetramethylindodicarbocyanine (DiD), and incubated with C2C12 myotubes undergoing differentiation. Quantitative fluorescence imaging revealed a significantly higher uptake of Mymk-LNP 4 compared to non-functionalized LNPs, suggesting that Myomaker improves nanoparticle-cell interactions (Figure S5). We then evaluated the potency of mRNA transfection using Mymk-LNP 4 with varying Myomaker-to-lipid incorporation ratios (0.25%, 1%, 5%). TdTomato mRNA was encapsulated into LNPs at a fixed lipid-to-mRNA mass ratio of 20:1 using a microfluidic mixing approach [46]. All Mymk-LNP formulations retained uniform particle size, a low PDI, and high encapsulation efficiencies (Figure S6). Differentiating C2C12 myotubes were treated with 200 ng of tdTomato mRNA-loaded LNPs per well in a 96-well plate to assess mRNA transfection efficiency. Among the tested formulations, Mymk-LNP 4 demonstrated higher tdTomato expression and transfection efficiency, significantly outperforming non-functionalized LNP (Figure 2a,b). These results were further supported by quantitative fluorescence analysis, where all the Mymk-LNP 4 groups exhibited significantly greater tdTomato fluorescence intensity compared to the control LNPs, demonstrating that fusogen functionalization enhances tdTomato mRNA transfection efficiency in differentiating myotubes (Figure 2c).

To establish a benchmark for transfection efficiency, we compared Mymk-LNP 4 to MC3-LNPs, a gold-standard LNP formulation consisting of DLin-MC3-DMA ionizable lipid, DSPC, cholesterol, and DMG-PEG2000 (50/10/38.5/1.5, mol/mol). Luciferase (Luc) mRNA was encapsulated into both LNP formulations with high encapsulation efficiency (Figure S7a). A dose response study in C2C12 myotubes showed that both formulations exhibited minimal cytotoxicity (Figure S7b). However, Mymk-LNP 4 exhibited significantly higher bioluminescence intensity than MC3-LNPs at all tested doses, indicating superior mRNA transfection efficiency (Figure S7c).

To further demonstrate the advantage of our Mymk-LNP 4 formulation in skeletal muscle delivery, we compared its in vitro transfection efficiency with SM102-LNPs, a commercially approved formulation for intramuscular vaccines, consisting of SM102 ionizable lipid, DSPC, cholesterol, and DMG-PEG2000 (50/10/38.5/1.5, mol/mol). Mymk-LNP 4 and SM102-LNP exhibited minimal cytotoxicity in transfecting C2C12 myotubes (Figure S8a). However, when quantifying the Luc protein expression following Luc mRNA delivery, Mymk-LNP 4 transfected myotubes showed a substantially higher bioluminescence signal compared to those transfected with SM102-LNP, indicating the better performance of Mymk-LNP 4 in transfecting muscle cells. Notably, the incorporation of Myomaker proteins into SM102-LNPs resulted in a nearly three-fold increase in transfection efficiency in C2C12 myotubes, which further highlights the modular nature of this concept and Myomaker’s functional role in enhancing skeletal muscle-selective mRNA delivery (Figure S8b). Finally, other LNP formulations (**LNPs 1–3**), with or without Myomaker, were evaluated as controls. Among them, only **LNP 3** exhibited a slight increase in bioluminescence intensity after Myomaker conjugation, while **LNP 1** and **LNP 2** showed negligible changes, suggesting the specific lipid composition of LNPs influences Myomaker-mediated transfection enhancement (Figure S9).

### 2.3 ∣ Fusogen-Engineered LNPs Improve Selectivity of mRNA Delivery to Myotubes

Given that Myomaker expression is required on both cells to facilitate membrane fusion [32], we investigated whether Mymk-LNP 4 selectively enhances mRNA transfection in differentiating myotubes while minimizing uptake in non-expressing cells, such as myoblasts and fibroblasts, which lack Myomaker on their surfaces. To assess cell-type selectivity, Luc mRNA was encapsulated into LNPs, and transfection outcomes were evaluated in C2C12 differentiating myotubes, C2C12 proliferating myoblasts, and 10T½ fibroblasts. Mymk-LNP 4 (0.25% and 1%) demonstrated significantly higher Luc protein expression in myotubes compared to LNPs without Myomaker. In contrast, Mymk-LNP 4 (5%) showed reduced transfection efficiency, suggesting that an excessive Myomaker-to-lipid ratio may hinder delivery efficiency or cellular uptake (Figure 2d). Importantly, in myoblasts and fibroblasts, Mymk-LNP 4 (0.25%) displayed no significant difference in transfection efficiency, while higher Myomaker ratios (1% and 5%) led to a reduction in transfection efficiency (Figure 2e,f). When transfection outcomes across all cell types were plotted together, it became evident that Mymk-LNP 4 selectively transfected myotubes while exhibiting minimal uptake in myoblasts and fibroblasts (Figure 2g). These trends were consistent across alternative Mymk-LNP formulations with different DOTAP ratios (20 and 40 wt%), where Myomaker functionalization did not enhance transfection in the cell types with little or no Myomaker expression (Figure S10).

To further study Myomaker’s mechanism in membrane fusion, we assessed Mymk-LNP 4’s fusion efficiency against plasma membrane-mimicking liposomes (PMMLs) using a Förster Resonance Energy Transfer (FRET) assay dependent on a pair of fluorescent probes. This is a process in which an excited donor fluorophore transfers energy non-radiatively to a nearby acceptor fluorophore when the donor emission spectrum overlaps with the acceptor excitation spectrum. The intact PMMLs were labeled by a fluorophore pair consisting of NBD (donor) and Rhodamine (Acceptor), resulting in a strong FRET signal because of their close association. Upon fusing with LNPs, lipid mixing increased the average distance between the two probes, leading to a reduction in the FRET signal and concomitant increased NBD fluorescence. Therefore, the efficiency of membrane fusion can be reflected by measuring NBD fluorescence. Compared to LNP 4, Mymk-LNP 4 exhibited a nearly twofold increase in the membrane fusion efficiency, which was dependent on Myomaker- mediated membrane-membrane fusion (Figure 2h). Collectively, these findings demonstrate that Mymk-LNP 4 exhibits selective transfection in differentiating myotubes expressing Myomaker, supporting its potential as a muscle-enhanced mRNA delivery platform.

### 2.4 ∣ Fusogen-Engineered LNPs Enhance mRNA Delivery to Skeletal Muscle Undergoing Fusion In Vivo

To evaluate the in vivo transfection efficiency of Mymk-LNPs in skeletal muscle, we selected three animal models in which muscle fusion is actively occurring and Myomaker is highly expressed, potentially facilitating Mymk-LNP-mediated delivery. We assessed delivery of Luc mRNA at a dose of 0.1 mg/kg in Mymk-LNP 4 and compared it with mRNA-loaded LNPs lacking Myomaker by several administration routes. First, we assessed mRNA transfection via facial vein injection in neonatal mice at postnatal day 2 (P2), a stage when Myomaker expression is elevated during early postnatal skeletal muscle growth [35] (Figure 3a). At 6 h post-injection, Luc protein expression in skeletal muscle was 4.2-fold higher in the Mymk-LNP 4-treated group compared to LNP-treated control group (Figure 3a). Bioluminescence analysis of major organs—including the heart, lungs, liver, spleen, and kidneys—revealed no significant differences between these two groups, further supporting the conclusion that Myomaker facilitates targeted enrichment of Mymk-LNP 4 in neonatal skeletal muscle without affecting systemic biodistribution (Figure S11).

Next, we examined systemic mRNA delivery in a chemically induced muscle injury model, which induces muscle regeneration and a fusion process [37]. Muscle injury was induced by injecting BaCl_2_ solution into the tibialis anterior (TA) muscle, followed by a 4-day recovery period before intravenous (IV) administration of Mymk-LNP 4 or control LNPs encapsulating Luc mRNA (Figure 3b). At 6 h after LNP administration, the TA muscles were harvested and analyzed via an in vivo imaging system. Bioluminescence imaging revealed strong Luc signals in the TA muscles of injured mice treated with Mymk-LNP 4. Quantitative analysis confirmed significantly higher transfection efficiency in the Mymk-LNP 4 group compared to LNPs lacking Myomaker. In contrast, mRNA transfection efficiency was similar between Mymk-LNP 4 and LNPs in uninjured TA muscle, suggesting Myomaker’s role in enriching mRNA delivery in regenerating muscles undergoing fusion. (Figure 3b). In a quantitative biodistribution analysis, Mymk-LNP 4 also showed higher accumulation in injured muscle compared with the LNP 4 control, suggesting a Myomaker-mediated fusion mechanism, consistent with the in vitro results (Figure S12). Next, we evaluated local mRNA transfection through intramuscular (IM) injection in the same BaCl_2_ muscle injury model (Figure 3c). Consistent with previous findings, Mymk-LNP 4 resulted in enhanced mRNA-mediated protein expression in injured muscle compared to uninjured muscle or injured muscle injected with LNPs lacking Myomaker (Figure 3c). Bioluminescence analysis of major organs revealed negligible Luc activity signals in the heart, lungs, liver, spleen, and kidneys, indicating that Myomaker functionalization of LNPs restricted their expression to the muscle and prevented systemic leakage following local injection (Figure S13). Notably, Luc activity signals observed after IM injection (Figure 3c) were substantially stronger than those observed after IV administration at the same dose of 0.1 mg/kg (Figure 3b), highlighting the effectiveness of local delivery for muscle-targeted mRNA expression.

### 2.5 ∣ Fusogen-Engineered LNPs Enabled Cre-Mediated Recombination in Regenerating Skeletal Muscle

To assess the potential of fusogen-engineered LNPs for Cre-mediated recombination in skeletal muscle, we utilized genetically engineered Ai14 tdTom reporter mice that contain a stop cassette flanked by LoxP sites that prevent tdTomato expression. Successful delivery of Cre mRNA via LNPs enables Cre protein expression, leading to excision of the stop cassette and induction of tdTomato fluorescence exclusively in transfected cells (Figure 4a). Similar to the Luc mRNA delivery, BaCl_2_ was used to induce muscle injury. Cre mRNA-loaded Mymk-LNP 4 (0.005, 0.05, 0.25 and 1 mg/kg) was intramuscularly injected into BaCl_2_-injured muscles four days post injury, and tdTomato expression was analyzed seven days after LNPs injection. The tdTomato fluorescence showed a dose-dependent increase in injured skeletal muscles, with maximal expression observed at 1 mg/kg (Figure 4b–d). Notably, negligible Cre-mediated recombination was found in non-target organs, such as the liver and lungs, highlighting the ability of Mymk-LNPs to reduce off-tissue effects—a limitation of many LNP designs [47] (Figure S14).

Further, mice treated with Mymk-LNP 4 exhibited significantly higher tdTomato fluorescence intensity in TA muscles than those receiving non-functionalized LNPs and commercial MC3-LNPs (Figure 4e). Fluorescence and confocal imaging confirmed the extensive presence of tdTomato^+^ myofibers in the Mymk-LNP 4-Cre group (Figure 4f,g), with transfection efficiencies of 32.5% in TA—markedly higher than those achieved with non-functionalized LNPs (15.7%) and MC3-LNPs (6.0%) (Figure 4h). Quantification of relative fluorescence intensity further corrob-orated these findings (Figure 4i), demonstrating the enhanced efficiency of Mymk-LNP 4 for Cre mRNA-mediated DNA recombination in regenerating skeletal muscle.

### 2.6 ∣ Tolerability of Fusogen-Engineered LNPs

To assess the biocompatibility and potential systemic toxicity of Mymk-LNP 4, we evaluated serum biomarkers and histopathology following both IV and IM administration. Mice received Mymk-LNP 4 or PBS via IV and IM injections, and toxicity markers were analyzed both 24 h and 1 week post-injection. Serum biochemistry analysis revealed no significant changes in Alanine aminotransferase (ALT), Aspartate aminotransferase (AST), Blood urea nitrogen (BUN), or Creatinine (CREA) levels between Mymk-LNP 4-treated and control groups, indicating no detectable hepatic or renal toxicity (Figure 5a,b). To further evaluate for a potential inflammatory response after Mymk-LNP 4 treatment, we measured the serum levels of different pro-inflammatory cytokines, including TNF-*α*, IL-1*β*, and IL-6, both 24 h and 1 week after either IV or IM injection. Compared with healthy mice administered with PBS, no statistically significant difference was observed for any of the cytokine levels in the Mymk-LNP 4-treated mice (Figure 5c,d). Finally, the heart, lung, liver, spleen, and kidneys were also collected and processed by hematoxylin and eosin (H&E) staining for histopathological analysis. We found that the overall structural integrity and immune infiltration of all the tissues in the Mymk-LNP 4-treated mice were nearly identical as compared to those in healthy mice (Figure 5e,f). Together, these results demonstrate that Mymk-LNPs exhibit excellent biocompatibility with no significant toxicity following systemic or localized administration, supporting their potential as a safe platform for muscle-targeted mRNA therapeutics.

## 3 ∣ Discussion

In this study, we developed protein-functionalized LNPs that enrich delivery to skeletal muscle. The fusogen Myomaker was integrated into various LNP formulations to facilitate muscle cell fusion. Optimized Mymk-LNPs, composed of four traditional lipid components and the cationic lipid DOTAP, exhibit high surface protein integration efficiency with the correct Myomaker orientation. These unique features enabled efficient mRNA delivery to muscle fusion protein-expressing cells in vitro and selectively transfected myotubes over non-Myomaker expressing cells (e.g., myoblasts and fibroblasts). In vivo, both systemic and local administration of Mymk-LNPs resulted in higher and more durable Luc expression in skeletal muscle compared to non-functionalized LNPs across three animal models, where muscle fusion is activated and fusogens are highly expressed. In a muscle-injured Ai14 reporter mouse model, Mymk-LNP 4 achieved dose-dependent and sustained target protein expression in TA muscles, demonstrating enhanced dispersion and multiple-fold higher signal compared to both control LNPs and clinically approved MC3-LNPs. Moreover, IV and IM administration of Mymk-LNP 4s was shown to be safe, further supporting their translational potential.

In summary, this Mymk-LNP platform introduces a promising strategy for muscle-specific mRNA delivery that differs from previous conventional ligand-mediated targeting approaches, as it seeks to exploit a fusogen-mediated membrane fusion mechanism to improve non-viral nucleic acid delivery, serving as a proof-of-concept for advancing LNP-mediated gene therapy for muscle disorders. In the future, it will be of interest to test whether additional modifications of the LNP formulation can further enhance muscle-specific delivery of LNPs. For example, inclusion of the fusogenic micropeptide Myomixer (also known as Myomerger or Minion) or the muscle-targeting peptide derived from the AAV capsid might further enhance specificity and efficiency of muscle delivery [48–50]. Alternatively, antibodies or ligands directed against muscle-specific epitopes could also be explored as targeting moieties to broaden the applicability of this platform. Moreover, systematic comparison of Mymk-LNPs with existing muscle-targeting strategies, such as AAV-MHCK7 or muscle-homing peptides (e.g., M12), will be valuable in the future. Looking ahead, future studies will also be directed toward evaluating the therapeutic efficacy of Mymk-LNPs in disease-relevant models for Duchenne muscular dystrophy following IM and IV administration. These investigations will be valuable for further development of Mymk-LNPs for treating severe muscle disorders.

## 4 ∣ Methods

### 4.1 ∣ Materials

5A2-SC8 was synthesized by following published procedures [51]. DOPE, DODAP, 18PA, DOTAP, and DSPC were obtained from Avanti Polar Lipids. Cholesterol was purchased from Sigma–Aldrich. DMG-PEG2000 was purchased from NOF America Corporation. HEPES buffer (1 M) was purchased from Thermo Fisher. Cas9 mRNA, firefly luciferase mRNA, tdTomato mRNA, and Cre mRNA were synthesized by in vitro transcription. The ONE-Glo + Tox Luciferase Reporter assay kit was purchased from Promega Corporation. Pur-A-Lyzer Midi Dialysis Kit (WMCO, 3.5 kDa) was purchased from Millipore Sigma. Pierce BCA Protein Assay Kit and Quant-iT RiboGreen RNA Assay Kit were purchased from Thermo Fisher.

### 4.2 ∣ Myomaker Expression and Purification

The complementary DNA (cDNA) of mouse Myomaker (Protein ID NP_079652.1) was cloned into pFastBac1 with a N-terminal Flag-tag. Recombinant Myomaker was expressed in Spodoptera frugiperda (Sf9) cells using the baculovirus expression system, following the manufacturer’s protocol (Thermo Fisher). Protein expression was induced by infecting Sf9 cells with recombinant baculovirus, and cells were harvested 48 h post-infection by centrifugation at 2000 × g for 10 min at 4°C. The cell pellet was resuspended in lysis buffer (20 mM HEPES, pH 7.5, 150 mM NaCl) supplemented with 1 mM phenylmethylsulfonyl fluoride (PMSF) and 5 μg/mL leupeptin, and disrupted by sonication on ice. The lysate was clarified by low-speed centrifugation (4000 × *g*, 10 min, 4°C) to remove cell debris. The resulting supernatant was incubated with 1% (w/v) lauryl maltose neopentyl glycol (LMNG) (Anatrace, NG310) and 0.1% cholesteryl hemisuccinate (CHS) (Anatrace, CH210) for 1 h at 4°C to solubilize membrane-associated proteins. Insoluble material was removed by ultracentrifugation (100 000 × *g*, 30 min, 4°C), and the supernatant was incubated with Flag M2 affinity resin (Sigma–Aldrich, A2220) for 2 h at 4°C with gentle mixing. The resin was washed twice with buffer A (20 mM HEPES, pH 7.5, 150 mM NaCl, 0.01% (w/v) LMNG, and 0.001% CHS) to remove non-specifically bound proteins. Myomaker was eluted with buffer A supplemented with 100 μg/mL 3×Flag peptide (Sigma–Aldrich) and further concentrated using Amicon Ultra centrifugal filters (MWCO 50 kDa, Millipore). The protein was subsequently purified using Superose 6 Increase 10/300 GL size-exclusion chromatography (GE Healthcare) equilibrated in buffer B (20 mM HEPES, pH 7.5, 150 mM NaCl, 0.001% LMNG, 0.0001% CHS, and 0.0025% glyco-diosgenin (GDN, Anatrace GDN101)). The eluted protein fractions were pooled, concentrated, flash-frozen in liquid nitrogen, and stored at −80°C for further experiments.

### 4.3 ∣ Lipid Nanoparticle Formulation

**LNP 1** consisted of ionizable lipid (5A2-SC8), helper lipid (DOPE), cholesterol, and PEGylated lipid (DMG-PEG2000) at a molar ratio of 36/20/40/4. Additional LNP formulations (**LNP 2** to **LNP 4)** were designed by incorporating the lipids (18PA, DODAP, and DOTAP) while maintaining the fixed molar ratio of the four primary lipids. The molar compositions of 5A2-SC8/DOPE/cholesterol/DMG-PEG2000/additional lipids were 32.4/18/36/3.6/10 for **LNP 2**, 28.8/16/32/3.2/20 for **LNP 3**, and 25.2/14/28/2.8/30 for **LNP 4**. All lipid components were dissolved in anhydrous ethanol, while mRNA (Luc, tdTomato, or Cre recombinase) was dissolved in 10mM citrate buffer (pH 4.0). The aqueous and ethanol phases were rapidly mixed at a 3:1 aqueous-to-ethanol ratio using either small-scale pipette-based rapid mixing or T-mixing for large-scale production. The total lipid-to-mRNA weight ratio was set at 20:1. LNPs were immediately subjected to dialysis against PBS using a 3.5 kDa MWCO Pur-A-Lyzer Midi dialysis kit under gentle stirring at 100 rpm at 4°C for 2 h to remove excess ethanol and unencapsulated mRNA. The resulting LNPs were characterized for size, polydispersity index (PDI), surface charge, and encapsulation efficiency using dynamic light scattering (DLS, Malvern Zetasizer Nano ZS) and Quant-iT RiboGreen RNA assay kit, ensuring high-quality mRNA-loaded nanoparticles for downstream applications.

### 4.4 ∣ Mymk-LNP Construction and Characterization

Mymk-LNPs were fabricated using a post-incubation surface modification method [44]. After dialysis purification, freshly synthesized LNPs were transferred to 20 mM HEPES and incubated with the recombinant Myomaker protein at various Myomaker-to-lipid weight ratios (0.25%, 1%, and 5%) at room temperature for 1 h. Following incubation, Mymk-LNPs were concentrated and purified using a 100 kDa Ultra filter tube (Amicon, UFC810096) according to the manufacturer’s instructions to remove unbound protein.

The final Mymk-LNP size, polydispersity index (PDI), and zeta potential were assessed using dynamic light scattering (DLS, Malvern Zetasizer Nano ZS, He-Ne laser, *λ* = 632 nm) to ensure uniformity and particle stability. The mRNA encapsulation efficiency was measured by Quant-iT RiboGreen RNA assay kit following the manufacturer’s protocol. To examine the long-term storage stability, both LNP 4s and Mymk-LNP 4s were incubated in PBS at 4°C for 7 days. The changes in nanoparticle size and encapsulation efficiency were evaluated using the DLS and Quanti-iT RiboGreen RNA assay kits, respectively. To confirm Myomaker protein conjugation efficiency, Mymk-LNPs were analyzed using the bicinchoninic acid (BCA) protein assay (Pierc BCA Protein Assay Kit, Thermo Fisher). Briefly, unbound protein in the flow-through of Mymk-LNP 4s after concentration and purification via 100 kDa Amicon Ultra filter tubes, was quantified by incubation with BCA reagent at 37°C for 30 min. Absorbance was measured at 562 nm using Infinite F/M200 Pro microplate reader (Tecan), and conjugation efficiency was calculated as [(Total input protein-unbound protein)/Total input protein] × 100%.

Western blotting was performed to confirm the presence of Mymk proteins on the LNP. The LNP samples, with or without Mymk, were lysed in RIPA buffer and diluted in 4× sample buffer (Bio-Rad, 1610747) containing 2-mercaptoethanol. Electrophoresis was conducted at 70 V for 20 min followed by 120 V for 45 min. Proteins were then transferred to a polyvinylidene difluoride (PVDF) membrane using the Trans-Blot Turbo Transfer System (Bio-Rad) at 25 A for 8 min. The membrane was incubated overnight at 4°C with mouse anti-FLAG M2 antibody (Sigma–Aldrich, F1804) in 5% non-fat milk, followed by a 1 h incubation at room temperature with goat anti-mouse horseradish peroxidase (HRP) conjugated antibody (Bio-Rad, 1706516). The detection was performed using Western Blotting Luminol Reagent (Santa Cruz, sc-2048). Furthermore, to evaluate the serum stability, Mymk-LNP 4s were incubated in 50% mouse serum for 37°C for 0, 0.25, 0.5, 1, and 24 h, followed by purification of unbound Myomaker using Amicon Ultra Centrifugal Filter tubes (100 kDa). The incorporation of Myomaker on LNP 4s after serum incubation was assessed by Western blot.

### 4.5 ∣ Transmission Electron Microscopy

To confirm Myomaker conjugation on LNP surfaces, immuno-gold labeling followed by transmission electron microscopy (TEM) was performed. Mymk-LNP 4 and control LNPs (without Myomaker) were incubated with anti-Myomaker primary antibodies (1:100 in PBS with 1% BSA) at 4°C for 1 h. Following three washes with PBS containing 1% BSA, samples were incubated with 12-nm colloidal gold-conjugated secondary antibodies (Jackson ImmunoResearch, 1:200) at 4°C for 1 h. After labeling, samples were fixed with 2% glutaraldehyde in PBS for 10 min.

Labeled LNPs were deposited onto a carbon-coated copper grid (400 mesh, Electron Microscopy Sciences), negatively stained with 1% uranyl acetate for 1 min, and allowed to air-dry. Imaging was performed using a JEOL-1400 Plus operated at 120 kV. A control sample of LNPs without Myomaker was processed under identical conditions to confirm specificity.

### 4.6 ∣ Cryogenic Transmission Electron Microscopy

Cryo-EM grids were prepared using a Mark IV Vitrobot (Thermo Fisher) under 100% humidity at 25°C. A 4 μL aliquot of fresh vesicle sample was applied to glow-discharged Quantifoil R1.2/1.3 300-mesh gold holey carbon grids (Quantifoil) and incubated for 10 s. Excess liquid was removed, and a second 4 μL aliquot was applied, followed by nominal blotting under zero force for 3 s. Grids were then vitrified by plunging into pre-cooled liquid ethane. Prepared grids were imaged on a 200 kV Glacios (Thermo Fisher) equipped with a K3 Summit direct electron detector (Gatan). Image acquisition was performed using SerialEM software at 45 000× magnification, with a defocus setting of −2.5 μm and an exposure time of 2 s.

### 4.7 ∣ Determination of Myomaker Protein Orientation by confocal Microscopy

To determine the orientation of Myomaker protein conjugated on the Mymk-LNP 4, immunofluorescence staining was performed. Mymk-LNP 4 was permeabilized with 1% Triton X-100/PBS, pH 7.4. Both permeabilized and unpermeabilized LNPs with Myomaker were incubated for 2 h on ice with rabbit anti-FLAG M2 antibody (Sigma–Aldrich, F7425) to recognize the extracellular N-terminus of Myomaker and 18G7 mouse anti-Mymk antibody [36] to recognize the intracellular region of Myomaker. The LNPs were then fixed with 4% paraformaldehyde (PFA) followed by an incubation of goat anti-rabbit Alexa Fluor 488 and goat anti-mouse Alexa Fluor 555 secondary antibodies for 1 h on ice. The samples were loaded onto *μ*-Slide 8-well chamber (ibidi) for imaging using a Zeiss LSM 800 confocal microscope.

### 4.8 ∣ In Vitro Transfection

To evaluate the mRNA transfection efficiency of Mymk-LNP 4, transfection studies were conducted in C2C12 myotubes, C2C12 myoblasts, and 10T½ fibroblasts. Cells were seeded in 96-well plates at a density of 1 × 10^4^ cells per well and maintained in Dulbecco’s Modified Eagle Medium (DMEM) supplemented with 10% fetal bovine serum (FBS) and 1% penicillin-streptomycin (Gibco, Thermo Fisher). C2C12 myoblasts were differentiated into myotubes by switching to differentiation media (DMEM containing 2% horse serum) for 3 days, with media changed every 48 h.

For cell selectivity studies, Luc mRNA-loaded Mymk-LNP 4 or a control group lacking Myomaker were applied to C2C12 myotubes, C2C12 myoblasts, and 10T½ fibroblasts, respectively, at a dose of 200 ng mRNA per well in 100 μL of DMEM. After 16 h, Luc activity and cytotoxicity were measured using the ONE-Glo + Tox kits using the recommended manufacturer’s standard protocol. To compare transfection efficiency across different LNP formulations (**Lipid 1–Lipid 3**), Luc mRNA-loaded LNPs were added to C2C12 myotubes at 100 ng mRNA per well, and Luc protein expression and cytotoxicity were quantified after 16 h using the same assay. For fluorescence-based transfection analysis, tdTomato mRNA-loaded Mymk-LNP 4 was delivered to C2C12 myotubes (200 ng mRNA per well), and tdTomato expression was visualized via Zeiss LSM 800 confocal microscopy. Cells were then lysed in lysis buffer (Promega, 50 μL per well) and fluorescence intensity was quantified under plate reader at excitation/emission wavelengths of 554 nm/581 nm.

### 4.9 ∣ In Vitro Membrane Fusion FRET Study

The membrane fusion efficiency of Mymk-LNP 4 and LNP 4 was investigated by tracking fusion events in vitro using plasma membrane-mimicking liposomes (PMMLs). To prepare PMMLs, DOPC, DOPE, NBD Lyso PE, Liss Rhod PE, SM, and cholesterol (20/18/1/1/20/30, mol/mol) were mixed in chloroform. After drying the mixture in a vacuum oven, the thin lipid film of PMMLs was rehydrated with 1X PBS (pH 7.5) containing Myomaker proteins (0.25% Myomaker-to-lipid weight ratios). For Myomaker incorporation, the suspension was sonicated in a bath sonicator for 15 mins, followed by incubation at room temperature for 1 h. The PMMLs were then extruded using Avanti’s mini extruder with a polycarbonate membrane size of 100 nm (Cytiva) to ensure homogeneity.

To measure membrane fusion, Mymk-LNP 4 or LNP 4 were incubated with PMMLs at LNP-to-liposome molar ratio of 10:1, for 2 h at room temperature. Fusion events were quantified by measuring the changes in NBD emission intensity (535 nm) as a result of decreasing FRET efficiency. The PMMLs only group and 2% Triton-X treated PMMLs were used as minimal and maximum NBD intensity, respectively. The membrane fusion efficiency was calculated as:

$$Membrane\;fusion\;effciency=\frac{FI_{\text{LNP}}-FI_{\text{Min}}}{FI_{\max}-FI_{\text{Min}}}\times 100$$

### 4.10 ∣ Animal Studies

All animal experiments were approved by the Institution Animal Care and Use Committees of The University of Texas Southwestern Medical Center and were consistent with local, state, and federal regulations as applicable. 6–8-week-old C57BL/6 mice were purchased from Charles River Laboratories. B6.Cg-Gt(ROSA)26Sortm14(CAG-tdTom)Hze/J mice (also known as Ai14 or Ai14(RCL-tdT) mice) were obtained from the Jackson Laboratory (007914) and bred to maintain the homozygous expression of the Cre reporter allele. To induce muscle injury and regeneration in vivo, mice were anesthetized with 2.5% Avertin (250 mg/kg; intraperitoneal injection). The skin over the target muscle was shaved, and then 1.2% BaCl2 was injected into the TA (30 μL) muscle. Mice were kept warm during recovery and then returned to their cage and were monitored daily post-injury.

### 4.11 ∣ In Vivo Luc mRNA Delivery

For the neonatal mice model, C57BL/6 mice (P2, postnatal day 2) were anesthetized on ice and then injected with Luc mRNA-loaded Mymk-LNP 4 (0.1 mg/kg) via the facial vein, while control mice received Luc mRNA-loaded LNP lacking Myomaker. At 6 h post-injection, Luc protein expression was measured via bioluminescence imaging using an AMI HTX in vivo imaging system (Spectral Instruments Imaging). Specifically, mice were anesthetized using 2% isoflurane, injected intraperitoneally with D-Luciferin substrate (150 mg/kg, GoldBio), and imaged 30 sec post-substrate injection. Bioluminescence intensity was quantified as total flux (photons/sec) using Aura Image Software (Spectral Instruments Imaging).

For targeted muscle mRNA delivery, a BaCl_2_-induced muscle injury model was used to mimic regenerating skeletal muscle. Adult C57BL/6 mice (6–8 weeks old) were anesthetized using 2.5% avertin, and 30 μL of 1.2% BaCl_2_ solution was injected into the TA muscle to induce localized injury. After a 4-day recovery period, mice received either IV or IM injection of 25 μL Luc mRNA-loaded Mymk-LNP 4 (0.1 mg/kg) into the TA muscle with or without injury. Control groups received Luc mRNA-loaded LNPs lacking Myomaker. Bioluminescence imaging was performed at 6 h post-injection, following the same protocol as described above.

### 4.12 ∣ In Vivo Cre mRNA Delivery

A BaCl_2_-induced muscle injury model was used to assess Cre-mediated recombination in regenerating skeletal muscle. C57BL/6 Ai14 reporter mice (6–8 weeks old) were anesthetized with 2% isoflurane, and 30 μL of 1.2% BaCl_2_ solution was injected into the TA muscle to induce localized injury. After a 4-day recovery period, Cre mRNA-loaded Mymk-LNP 4 (0.005, 0.05, 0.25, or 1 mg/kg in 25 μL PBS) were intramuscularly injected into the injured TA muscles while control groups received non-functionalized LNPs or PBS. At 7 days post-injection, tdTomato fluorescence expression was analyzed using AMI HTX in vivo imaging. Mice were anesthetized with 2% isoflurane, and fluorescence signals were captured using 570/620 nm excitation/emission filters. For ex vivo fluorescence analysis, mice were euthanized via CO_2_ asphyxiation and cervical dislocation, and TA, liver, and lung tissues were harvested. All tissues were fixed at 4°C for 2 h with 4% paraformaldehyde (PFA), followed by an overnight dehydration in 10% sucrose. The tissues were then further dehydrated in 18% sucrose at 4°C for 3 h before embedding. Muscle tissues were embedded in a 1:2 volume mixture of Gum Tragacanth powder (Sigma-Aldrich, G1128-500G) to Tissue Freezing Medium (General Data Healthcare, Inc.). All muscle samples were snap frozen in supercooled isopentane surrounding with liquid nitrogen. All the tissue blocks were stored at −80°C prior to sectioning. Ten-micrometer sections were prepared on a Leica CM3050 cryostat and mounted on glass slides. After DAPI staining, the slides were imaged using a Zeiss LSM 800 confocal microscope. The number of tdTomato-positive myofibers and mean intensity of tdTomato per field of view (FOV) was quantified using ImageJ software.

### 4.13 ∣ In Vivo Toxicity Evaluation

C57BL/6 mice (6–8 weeks old) were randomly assigned to receive either IV or IM injections of Mymk-LNP 4 or PBS as a control. For IV administration, 100 μL of Mymk-LNP 4 was injected via the tail vein, while for IM administration, 25 μL of Mymk-LNP 4 was delivered directly into the TA muscle. To ensure high systemic and localized exposure, a high mRNA dose 1 mg/kg for IV injections and 2.5 mg/kg for IM injections was used. At 24 h and 7 days post-injection, mice were anesthetized with 2% isoflurane, and whole blood was collected via submandibular venipuncture. Serum levels of ALT, AST, BUN, and CREA were measured at the UT Southwestern Metabolic Phenotyping Core to assess hepatic and renal function. Pro-inflammatory cytokine levels in serum, including TNF-*α*, IL-1*β*, and IL-6, were measured using ELISA (BioLegend) according to the manufacturer’s protocol. Additionally, heart, liver, spleen, lungs, and kidney were collected for histological analysis that was performed via H&E staining at the UT Southwestern Tissue Management shared resource to evaluate potential tissue damage or inflammation.

### 4.14 ∣ Statistical Analysis

Statistical analyses were performed using GraphPad Prism 10 and all relevant information is stated in the figure captions. In summary, all data are presented as mean + s.d. One-way ANOVA was used to determine the significance of comparisons of data in Figures 1–4. A two-tailed unpaired *t*-test was used to determine the significance of the biosafety studies in Figure 5. *P* values < 0.05 were considered statistically significant (* *p* < 0.05, ** *p* < 0.01, *** *p* < 0.001, **** *p* < 0.0001).

## Supplementary Material

Supporting materials

Additional supporting information can be found online in the Supporting Information section.

## Figures and Tables

**FIGURE 1 ∣:**
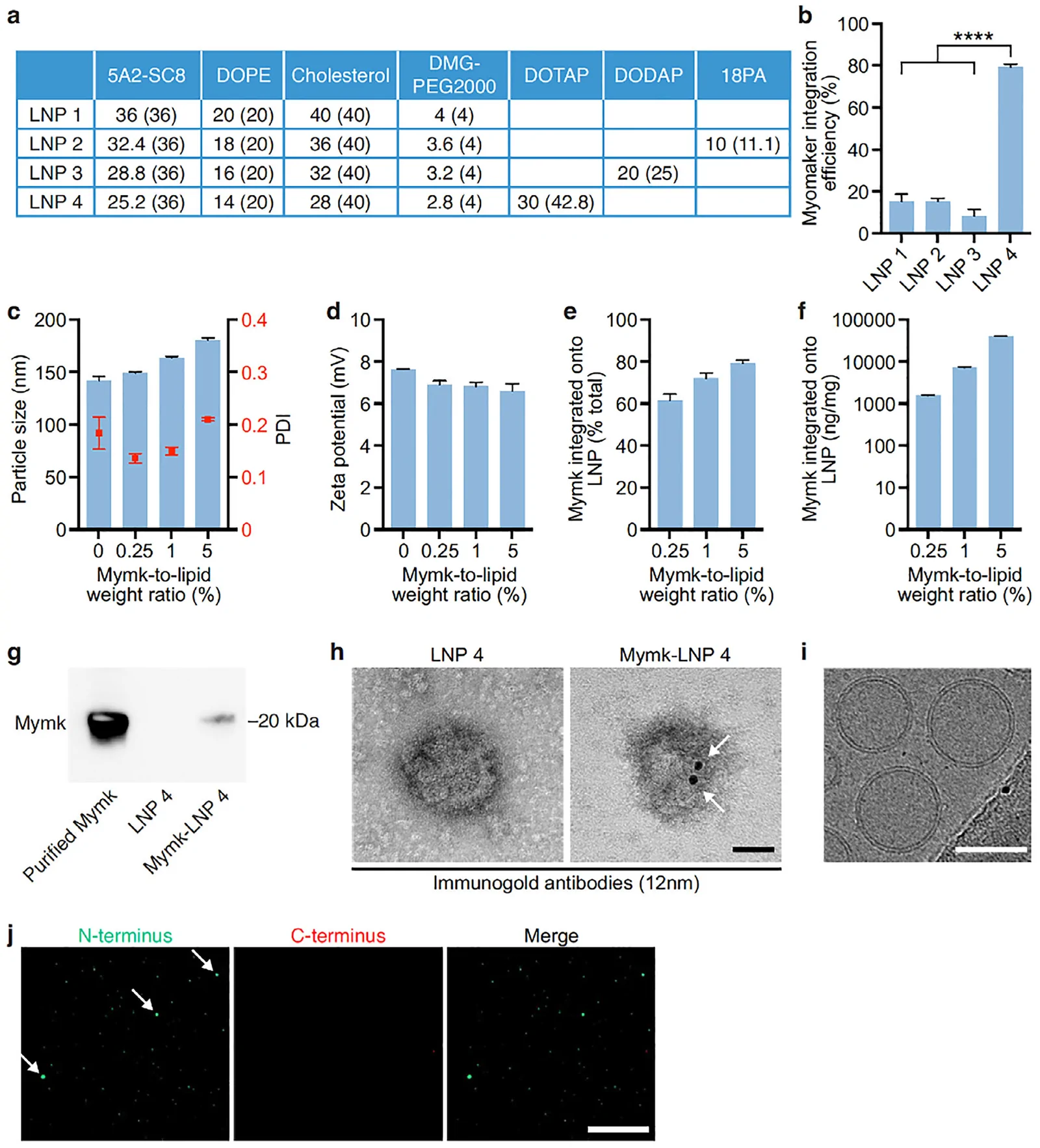
Characterization of Myomaker-functionalized LNPs (Mymk-LNPs). (a) Table listing the lipid composition for LNPs 1–4. The main values represent the molar percentages of each component, while the numbers in parentheses indicate the corresponding molar ratios. (b) Integration efficiency of Myomaker onto LNPs 1–4. (c) Particle size, PDI, and (d) zeta potential of LNP 4 after integration with various Myomaker inputs. (e) Myomaker integration efficiency and (f) amount of Myomaker on LNP 4 at different Myomaker input levels. (g) Western blot analysis of Myomaker on LNP 4 and Mymk-LNP 4, with purified Myomaker as a positive control. (h) Immunogold electron microscopy images of LNP 4 and Mymk-LNP 4 labeled with the indicated antibody. Arrowheads indicate Myomaker molecules labeled by immunogold on the surface of the LNPs. Scale bar, 50 nm. (i) Cryo-EM image showing the structure of Mymk-LNP 4. Scale bar, 100 nm. (j) Immunostaining of the Mymk-LNP 4 using an N-terminal FLAG antibody (green) and a C-terminal antibody (red) confirms the correct orientation of Myomaker after integration into LNP 4. Arrowheads indicate Myomaker N terminus labeled by the anti-FLAG antibody on the outer surface of the LNPs. Scale bar, 100 μm. In all datasets, n = 3; data are presented as mean + s.d. **** *p* < 0.0001. Statistical analysis was performed with one-way ANOVA.

**FIGURE 2 ∣:**
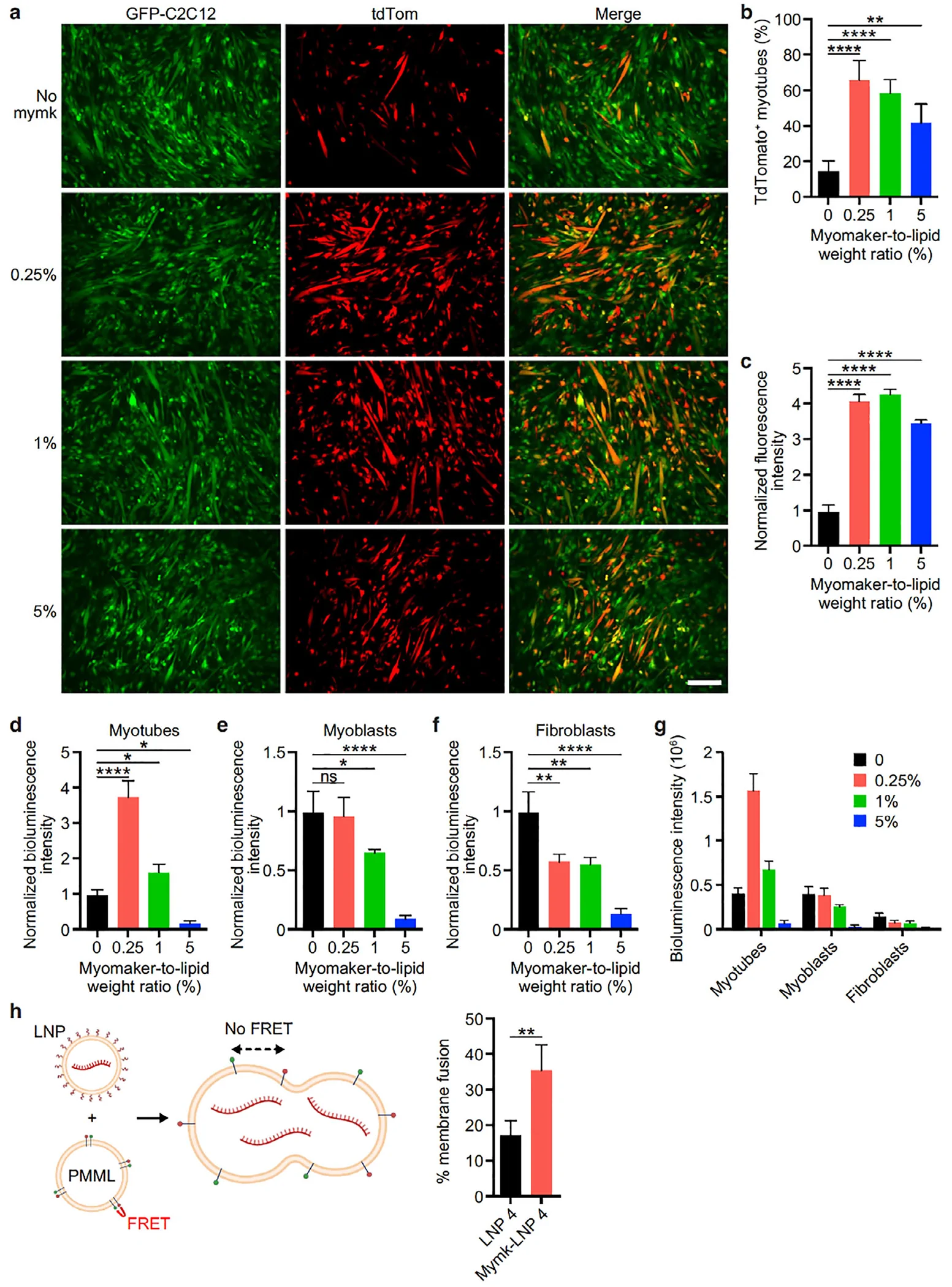
Mymk-LNPs enhance mRNA transfection efficiency and selectivity in myotubes. (a) Representative fluorescence images of differentiating C2C12 myotubes transfected with tdTomato mRNA-loaded Mymk-LNP 4 at varying Mymk-to-lipid ratios (0.25%, 1%, and 5%) compared to non-functionalized LNP. GFP (green) marks cell cytoplasm, tdTomato (red) indicates mRNA expression, and merged images highlight preferential transfection in myotubes. Scale bar, 200 μm. (b) Quantification of tdTomato expression in GFP-positive C2C12 myotubes shown in (a). (c) Fluorescence intensity analysis of tdTomato expression from a). Quantification of relative luciferase expression in (d) differentiating C2C12 myotubes, (e) proliferating C2C12 myoblasts, and (f) 10T½ fibroblasts. The bioluminescence intensity was normalized to the non-functionalized LNP group. (g) Comparative analysis of luciferase transfection efficiency across myotubes, myoblasts, and fibroblasts, demonstrating selective transfection in differentiating myotubes with minimal uptake in non-myogenic cells. (h) (Left) The schematic of membrane fusion measured by a Förster Resonance Energy Transfer (FRET) assay. Plasma membrane-mimicking liposomes (PMMLs) labeled with NBD (donor) and Rhodamine (acceptor) exhibited a strong FRET signal due to the proximity of the fluorophores. Upon fusion with LNPs, FRET decreased while NBD fluorescence increased and the NBD signal was used to reflect the efficiency of membrane fusion. (Right) Comparison of membrane fusion efficiency of Mymk-LNP 4 and LNP 4 against PMMLs, as measured by FRET 2 h post-incubation. In all datasets, n = 3; data are presented as mean + s.d. * *p* < 0.05, ** *p* < 0.01, **** *p* < 0.0001. Statistical analysis was performed with one-way ANOVA for (b–f), and student’s *t*-test for h).

**FIGURE 3 ∣:**
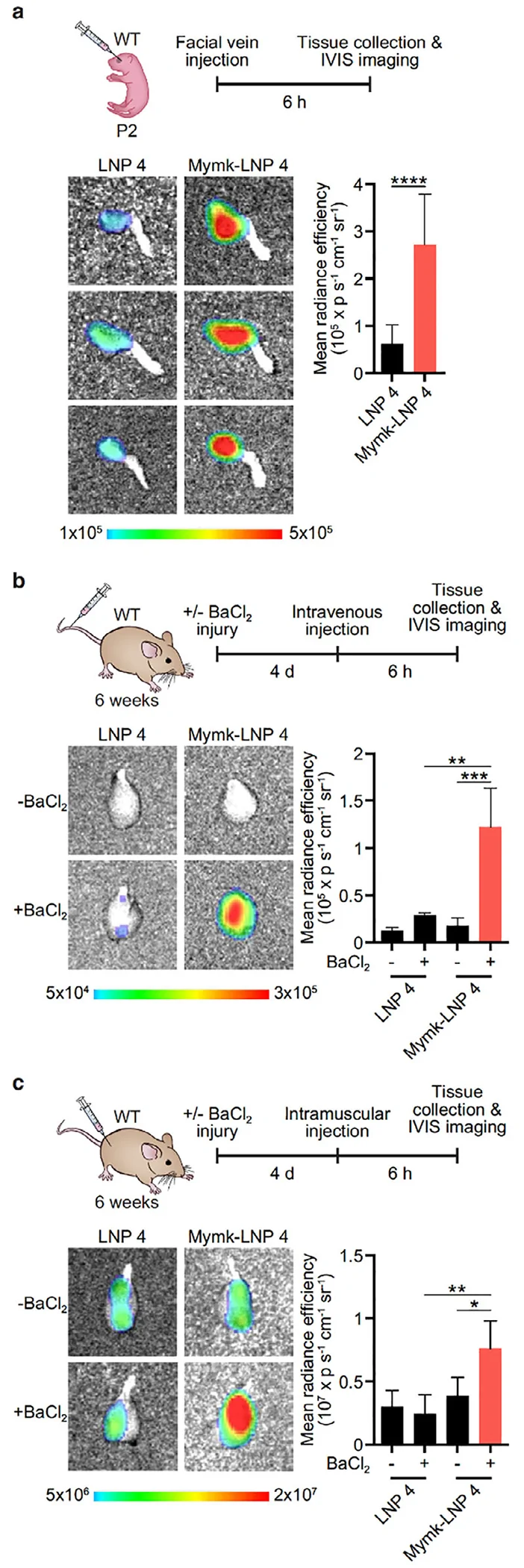
Mymk-LNPs enhance mRNA delivery to skeletal muscle in vivo. (a) Ex vivo bioluminescence imaging and quantification of excised skeletal muscle 6 h after facial vein injection of Mymk-LNP 4 or control LNPs at a luciferase mRNA dose of 0.1 mg/kg. Bioluminescence images and quantification of luciferase expression in injured or healthy tibialis anterior muscle 6 h after (b) intravenous or (c) intramuscular injection of Mymk-LNP 4 or control LNPs. In all datasets, n = 3; data are presented as mean + s.d. * *p* < 0.05, ** *p* < 0.01, *** *p* < 0.001, **** *p* < 0.0001. Statistical analysis was performed with a Student *t*-test for (a), and one-way ANOVA for (b, c).

**FIGURE 4 ∣:**
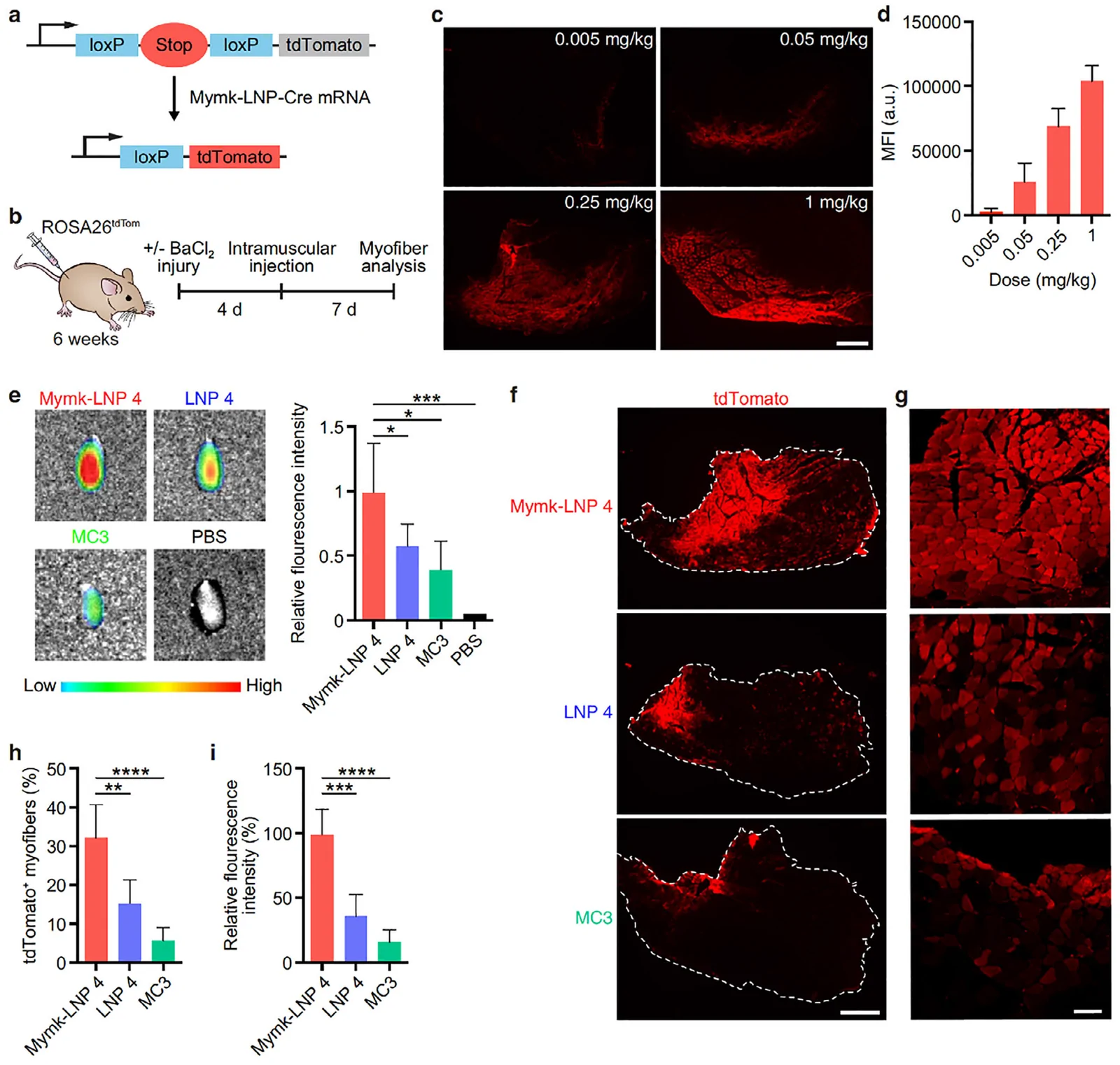
Fusion protein-engineered LNPs enable gene delivery and Cre-mediated recombination in injured skeletal muscle. (a) Schematic of Cre mRNA delivery enabling Cre protein expression and excision of the stop cassette, inducing tdTomato fluorescence in transfected cells. (b) Schematic of in vivo Cre mRNA delivery through intramuscular injection into tibialis anterior (TA) muscles 4 days post-injury, and tdTomato expression was analyzed 7 days later. (c) Dose-dependent tdTomato expression in skeletal muscle following intramuscular injection of Cre mRNA-loaded Mymk-LNP 4 (0.005, 0.05, 0.25, and 1 mg/kg) into BaCl_2_-injured TA muscles. Scale bar, 500 μm. (d) Quantification of tdTomato fluorescence intensity from (c). (e) Ex vivo bioluminescence imaging and quantification of tdTomato expression in TA muscle injected with Mymk-LNP 4, control LNPs, or MC3-LNPs, (f) Fluorescence imaging of tdTomato^+^ myofibers in TA muscles treated with Mymk-LNP 4, control LNPs, or MC3-LNPs. White dashed contour lines indicate the outer boundary of the TA muscle cross-section. Scale bar 500 μm. (g) High-magnification confocal images of tdTomato^+^ myofibers from (f). Scale bar, 100 μm. (h) Quantification of transfection efficiency and (i) relative fluorescence intensity in TA muscles. In all datasets, n = 3; data are presented as mean + s.d. * *p* < 0.05, ** *p* < 0.01, *** *p* < 0.001, **** *p* < 0.0001. Statistical analysis was performed with one-way ANOVA.

**FIGURE 5 ∣:**
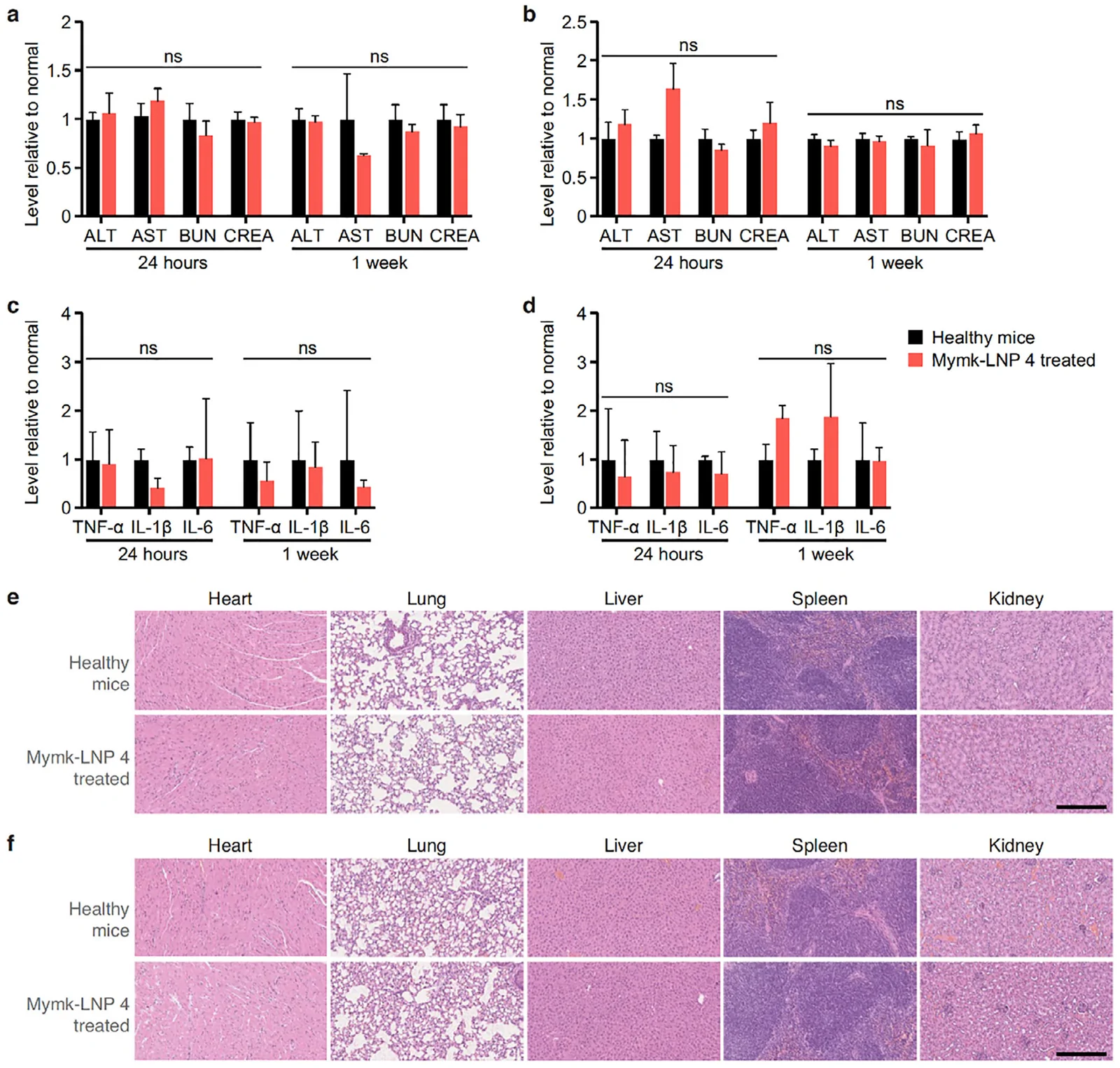
In vivo biosafety evaluation of Mymk-LNPs. 6–8-weeks-old male C57BL/6 mice were administered with PBS or Mymk-LNP 4 via intravenous (IV) (1.5 mg/kg mRNA) or intramuscular (IM) (1 mg/kg mRNA) injection. Short-term and long-term comprehensive serum chemistry panel analysis of PBS-treated healthy mice and mice administered with Mymk-LNP 4 through (a) IV and (b) IM injection. Pro-inflammatory cytokines, including TNF-*α*, IL-1*β*, and IL-6, were measured in the serum from PBS-treated healthy mice and mice administered with Mymk-LNP 4 through (c) IV and (d) IM injection. H&E stained histological sections of major organs collected 1 week after the treatment from PBS-treated healthy mice and mice administered with Mymk-LNP 4 through (e) IV and (f) IM injection. Scale bar, 250 μm. In all datasets, n = 3; data are presented as mean + s.d.; *n.s*. = not significant. Statistical analysis was performed with two-tailed unpaired multiple *t*-test.

## Data Availability

The data that support the findings of this study are available from the corresponding author upon reasonable request.
