# Supplementary material for: Myogenic Fusogen-Engineered Lipid Nanoparticles Enhance mRNA Delivery in Skeletal Muscle
Source: Adv Funct Mater. Author manuscript; Available in PMC 2026 Nov 14. (PMC13767189; doi:10.1002/adfm.78165)
Supplement: Supplementary file 1 [file NIHMS2221753-supplement-Supporting_materials.docx]

Supporting Information

**Myogenic Fusogen-Engineered Lipid Nanoparticles Enhance mRNA Delivery in Skeletal Muscle**

Fangyu Zhang^1,7^, Zhisheng Xu^2,7^, Yaou Duan^1,7^, Yehui Sun^1^, Shiying Wu^1^, Tao Long^3^, Mateusz Durbacz^2^, Li Zhang^5^, Yufen Xiao^1^, Yu-Chung Pien^2^, Erick D. Guerrero^1^, Zeru Tian^1^, Xu Wang^1^, Pratima Basak^1^, Sumanta Chatterjee^1^, Ning Liu^2^, Xiaochun Li^3,4^, Eric N. Olson^2,6*,^ Daniel J. Siegwart^1,*^

^1^Department of Biomedical Engineering, Department of Biochemistry, Simmons Comprehensive Cancer Center, Program in Genetic Drug Engineering, University of Texas Southwestern Medical Center, Dallas, TX, USA.

^2^Department of Molecular Biology, University of Texas Southwestern Medical Center, Dallas, TX, USA

^3^Department of Molecular Genetics, University of Texas Southwestern Medical Center, Dallas, TX, USA

^4^Department of Biophysics, University of Texas Southwestern Medical Center, Dallas, TX, USA

^5^Department of Physiology, University of Texas Southwestern Medical Center, Dallas, TX, USA

^6^Hamon Center for Regenerative Science and Medicine, University of Texas Southwestern Medical Center, Dallas, TX, USA

^7^These authors contributed equally: Fangyu Zhang, Zhisheng Xu, Yaou Duan

*Corresponding authors: [Daniel.Siegwart@UTSouthwestern.edu](mailto:Daniel.Siegwart@UTSouthwestern.edu); [Eric.Olson@utsouthwestern.edu](mailto:Eric.Olson@utsouthwestern.edu)

**This PDF file includes:**

Figs. S1 to S13

Figure S1. Schematic illustration of the dual-detection strategy used to verify the orientation of Myomaker proteins on the surface of LNPs.

Figure S2. Fluorescence-based secondary antibody detection of Myomaker orientation on different LNP formulations. Mymk-LNPs 1-3 exhibited markedly weaker and more sporadic fluorescence signals than LNP 4, indicating lower levels of Myomaker incorporation and random orientation. Arrowheads indicate the Myomaker N terminus labeled by the anti-FLAG antibody (green) or the Myomaker C terminus labeled by the endogenous antibody (red) on the outer surface of the LNPs. Scale bar, 50 μm.

Figure S3. Long-term storage stability study. a) Colloidal stability and b) mRNA encapsulation efficiency of LNP 4 and Mymk-LNP 4 over a period of 7 days in 1X PBS at 4 °C (n = 3; mean + SD).

**Figure S4**. Serum stability of Mymk-LNP 4s. Mymk-LNP 4s were incubated with 50% mouse serum for 0, 0.25, 0.5, 1, and 24 h. The integration of Myomaker on Mymk-LNP 4 was confirmed by western blot.

Figure S5. Comparison of uptake of DiD-labeled non-functionalized LNP 4 and Mymk-LNP 4 by differentiating C2C12 myotubes (n = 3; mean + s.d.).

Figure S6. Characterization of tdTom-mRNA-encapsulated Mymk-LNP 4 formulated with varying Myomaker-to-lipid incorporation ratios (0.25%, 1%, and 5%). a) Size, b) polydispersity index (PDI), and c) encapsulation efficiency of each formulation. In all datasets, n = 3; data are presented as mean + s.d.

Figure S7. Comparison of Mymk-LNP 4 and MC3-LNPs for Luc-mRNA delivery in differentiating C2C12 myotubes. a) Encapsulation efficiency of luciferase mRNA for both formulations (n = 3; mean + s.d.). b) Cell viability following treatments with increasing doses (20-400 ng/well) (n = 3; mean + s.d.). c) Comparison of dose-dependent Luc-mRNA expression, showing enhanced transfection efficiency of Mymk-LNP 4 compared to MC3-LNPs (n = 3; mean ± s.d.).

Figure S8. Comparison of Mymk-LNP 4, SM102-LNPs, and Mymk-SM102-LNPs for Luc-mRNA delivery in differentiating C2C12 myotubes. a) Cell viability following treatments with different LNP formulations at dosage of 200 ng/well (n = 3; mean + s.d.). b) Comparison of Luc-mRNA expression, showing enhanced transfection efficiency of Mymk-LNP 4 compared to SM102-LNPs and Mymk-SM102-LNPs. In all datasets, n = 3; data are presented as mean + s.d.

Figure S9. Evaluation of Luc-mRNA expression in differentiating C2C12 myotubes 16 hours after treatment with various luciferase mRNA-encapsulated LNP formulations (100 ng/well), with or without Myomaker functionalization. In all datasets, n=3; data are presented as mean + s.d.

Figure S10. Evaluation of cell-type–specific transfection efficiency of alternative Mymk-LNP formulations with varying DOTAP ratios (20 wt%, 30 wt% and 40 wt%). Luciferase mRNA expression in a) proliferating C2C12 myoblasts and b) 10T½ fibroblasts treated with Mymk-LNPs formulated at different Myomaker-to-lipid weight ratios (0.25%, 1%, and 5%). Minimal transfection was observed across all conditions, indicating that Myomaker functionalization does not enhance delivery in non-Myomaker–expressing cell types. In all datasets, n=3; data are presented as mean + s.d.

Figure S11. Bioluminescence imaging of major organs following facial vein injection of 0.1 mg/kg luciferase mRNA-encapsulated Mymk-LNP 4 or control LNP 4 in neonatal (P2) mice. Luciferase expression was assessed 6 hours post-injection in the heart, lungs, liver, spleen, and kidneys. No significant differences in organ signal intensity were observed between groups, indicating that Myomaker functionalization does not alter systemic biodistribution.

Figure S12. Biodistribution comparison between Mymk-LNP 4 and LNP 4 in mice with BaCl₂-induced muscle injury, shown as percentage injected dose per gram (%ID/g) of tissue. In all datasets, n = 3; data are presented as mean + s.d.

Figure S13. Bioluminescence imaging of major organs 6 hours after intramuscular injection of 0.1 mg/kg luciferase mRNA-encapsulated Mymk-LNP 4 or control LNP 4 in BaCl₂-induced muscle injury mice. Luciferase expression was assessed in the heart, lungs, liver, spleen, and kidneys. Minimal off-target expression was detected, indicating that Myomaker functionalization restricted mRNA delivery to muscle tissue without systemic leakage.

Figure S14. Ex vivo fluorescence imaging of tdTomato expression in TA muscle and major organs — including heart, lungs, liver, spleen, and kidney — following intramuscular injection of Mymk-LNP 4 delivering Cre mRNA at a dose of 0.5 mg/kg. Strong tdTomato signal was observed in the TA muscle, with negligible fluorescence in non-target organs, indicating tissue-specific gene editing and minimal off-target activity.
